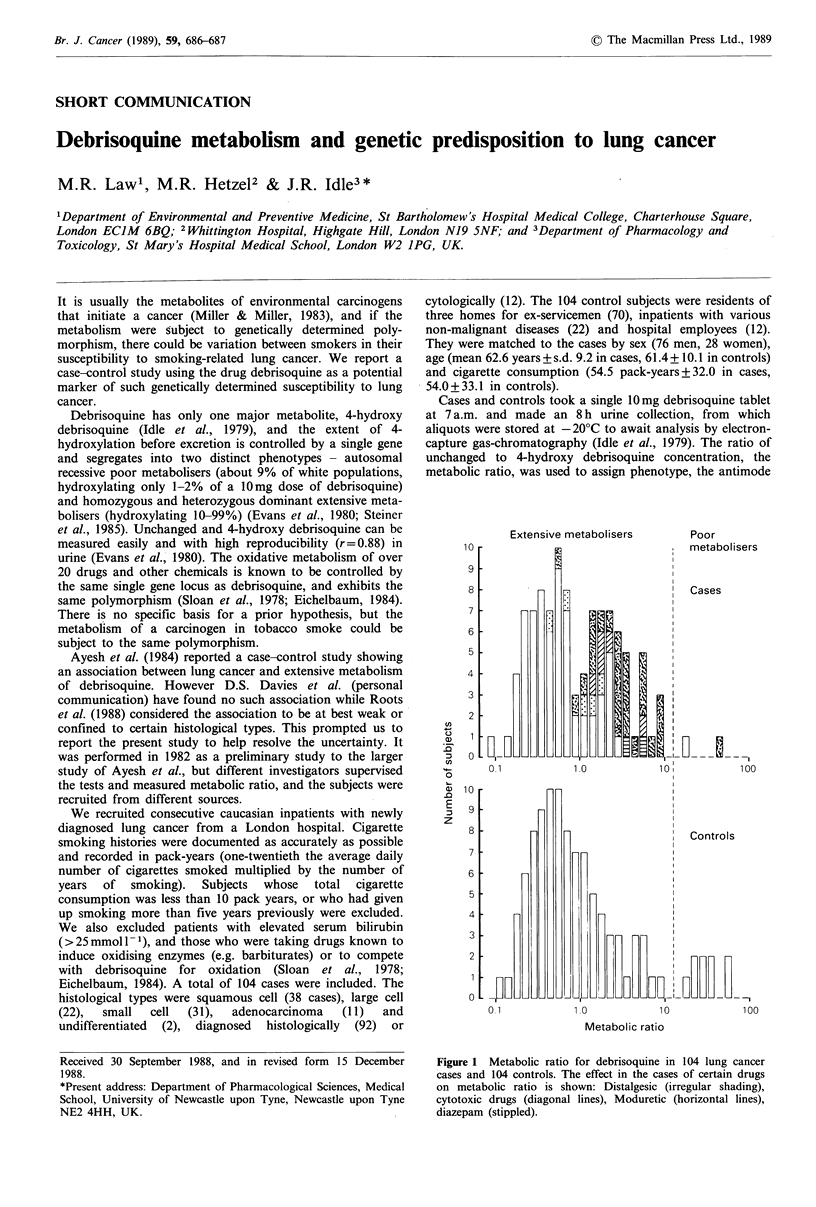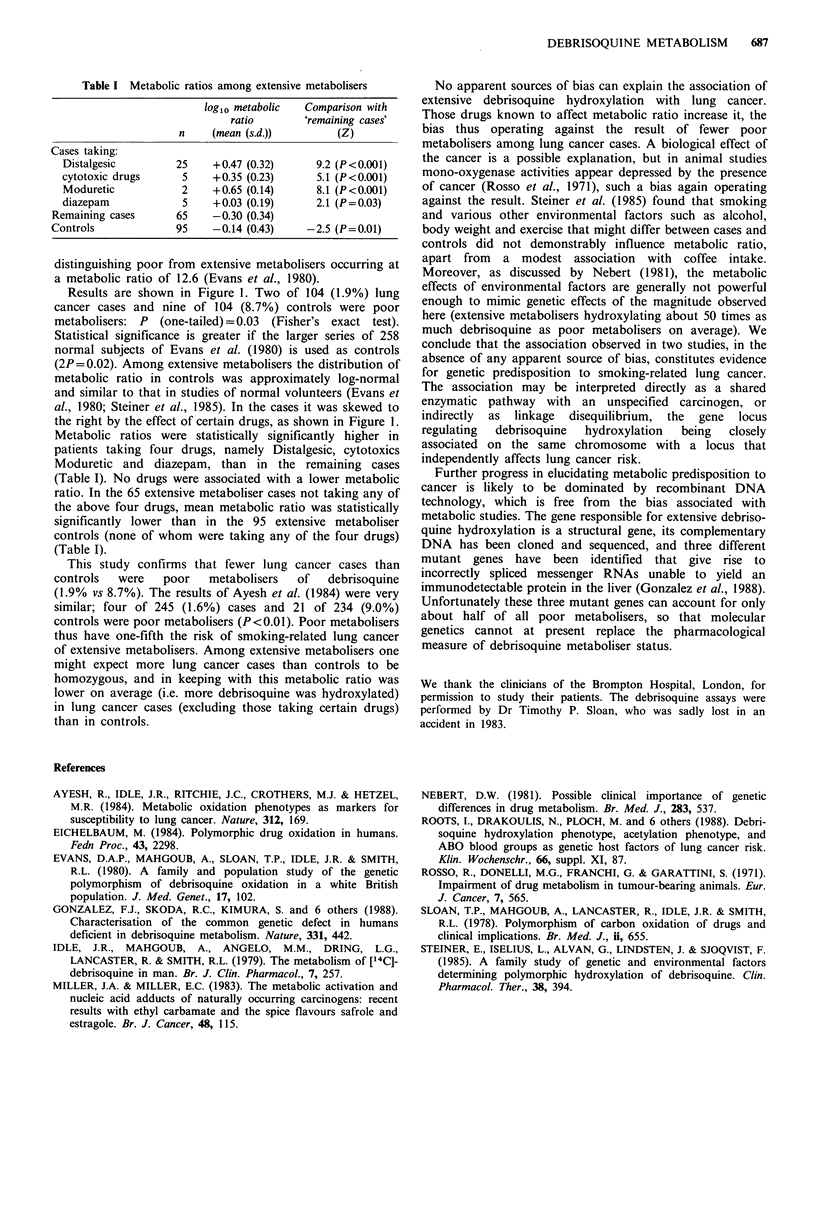# Debrisoquine metabolism and genetic predisposition to lung cancer.

**DOI:** 10.1038/bjc.1989.142

**Published:** 1989-05

**Authors:** M. R. Law, M. R. Hetzel, J. R. Idel

**Affiliations:** Department of Environmental and Preventive Medicine, St Bartholomew's Hospital Medical College, Charterhouse Square, London, UK.


Br. J. Cancer (1989), 59, 686–687 © The Macmillan Press Ltd., 1989

SHORT COMMUNICATION

### Debrisoquine metabolism and genetic predisposition to lung cancer

M.R. Law[1], M.R. Hetzel[2] & J.R. Idle[3]*

[1]*Department of Environmental and Preventive Medicine, St Bartholomew's Hospital Medical College, Charterhouse Square, London EC1M 6BQ;* [2]*Whittington Hospital, Highgate Hill, London N19 5NF; and* [3]*Department of Pharmacology and Toxicology, St Mary's Hospital Medical School, London W2 1PG, UK.*

It is usually the metabolites of environmental carcinogens that initiate a cancer (Miller & Miller, 1983), and if the metabolism were subject to genetically determined polymorphism, there could be variation between smokers in their susceptibility to smoking-related lung cancer. We report a case–control study using the drug debrisoquine as a potential marker of such genetically determined susceptibility to lung cancer.

Debrisoquine has only one major metabolite, 4-hydroxy debrisoquine (Idle *et al.*, 1979), and the extent of 4-hydroxylation before excretion is controlled by a single gene and segregates into two distinct phenotypes – autosomal recessive poor metabolisers (about 9% of white populations, hydroxylating only 1–2% of a 10 mg dose of debrisoquine) and homozygous and heterozygous dominant extensive metabolisers (hydroxylating 10–99%) (Evans *et al.*, 1980; Steiner *et al.*, 1985). Unchanged and 4-hydroxy debrisoquine can be measured easily and with high reproducibility ($r=0.88$) in urine (Evans *et al.*, 1980). The oxidative metabolism of over 20 drugs and other chemicals is known to be controlled by the same single gene locus as debrisoquine, and exhibits the same polymorphism (Sloan *et al.*, 1978; Eichelbaum, 1984). There is no specific basis for a prior hypothesis, but the metabolism of a carcinogen in tobacco smoke could be subject to the same polymorphism.

Ayesh *et al.* (1984) reported a case–control study showing an association between lung cancer and extensive metabolism of debrisoquine. However D.S. Davies *et al.* (personal communication) have found no such association while Roots *et al.* (1988) considered the association to be at best weak or confined to certain histological types. This prompted us to report the present study to help resolve the uncertainty. It was performed in 1982 as a preliminary study to the larger study of Ayesh *et al.*, but different investigators supervised the tests and measured metabolic ratio, and the subjects were recruited from different sources.

We recruited consecutive caucasian inpatients with newly diagnosed lung cancer from a London hospital. Cigarette smoking histories were documented as accurately as possible and recorded in pack-years (one-twentieth the average daily number of cigarettes smoked multiplied by the number of years of smoking). Subjects whose total cigarette consumption was less than 10 pack years, or who had given up smoking more than five years previously were excluded. We also excluded patients with elevated serum bilirubin ($>25\,\mathrm{mmol\,l^{-1}}$), and those who were taking drugs known to induce oxidising enzymes (e.g. barbiturates) or to compete with debrisoquine for oxidation (Sloan *et al.*, 1978; Eichelbaum, 1984). A total of 104 cases were included. The histological types were squamous cell (38 cases), large cell (22), small cell (31), adenocarcinoma (11) and undifferentiated (2), diagnosed histologically (92) or cytologically (12). The 104 control subjects were residents of three homes for ex-servicemen (70), inpatients with various non-malignant diseases (22) and hospital employees (12). They were matched to the cases by sex (76 men, 28 women), age (mean 62.6 years ± s.d. 9.2 in cases, 61.4 ± 10.1 in controls) and cigarette consumption (54.5 pack-years ± 32.0 in cases, 54.0 ± 33.1 in controls).

Cases and controls took a single 10 mg debrisoquine tablet at 7 a.m. and made an 8 h urine collection, from which aliquots were stored at −20°C to await analysis by electron-capture gas-chromatography (Idle *et al.*, 1979). The ratio of unchanged to 4-hydroxy debrisoquine concentration, the metabolic ratio, was used to assign phenotype, the antimode

**Figure 1** Metabolic ratio for debrisoquine in 104 lung cancer cases and 104 controls. The effect in the cases of certain drugs on metabolic ratio is shown: Distalgesic (irregular shading), cytotoxic drugs (diagonal lines), Moduretic (horizontal lines), diazepam (stippled).

Received 30 September 1988, and in revised form 15 December 1988.

*Present address: Department of Pharmacological Sciences, Medical School, University of Newcastle upon Tyne, Newcastle upon Tyne NE2 4HH, UK.

**Table I** Metabolic ratios among extensive metabolisers

| | *n* | *$log_{10}$ metabolic ratio (mean (s.d.))* | *Comparison with 'remaining cases' (Z)* |
|---|---|---|---|
| Cases taking: | | | |
| Distalgesic | 25 | +0.47 (0.32) | 9.2 ($P<0.001$) |
| cytotoxic drugs | 5 | +0.35 (0.23) | 5.1 ($P<0.001$) |
| Moduretic | 2 | +0.65 (0.14) | 8.1 ($P<0.001$) |
| diazepam | 5 | +0.03 (0.19) | 2.1 ($P=0.03$) |
| Remaining cases | 65 | −0.30 (0.34) | |
| Controls | 95 | −0.14 (0.43) | −2.5 ($P=0.01$) |

distinguishing poor from extensive metabolisers occurring at a metabolic ratio of 12.6 (Evans *et al.*, 1980).

Results are shown in Figure 1. Two of 104 (1.9%) lung cancer cases and nine of 104 (8.7%) controls were poor metabolisers: $P$ (one-tailed) = 0.03 (Fisher's exact test). Statistical significance is greater if the larger series of 258 normal subjects of Evans *et al.* (1980) is used as controls ($2P=0.02$). Among extensive metabolisers the distribution of metabolic ratio in controls was approximately log-normal and similar to that in studies of normal volunteers (Evans *et al.*, 1980; Steiner *et al.*, 1985). In the cases it was skewed to the right by the effect of certain drugs, as shown in Figure 1. Metabolic ratios were statistically significantly higher in patients taking four drugs, namely Distalgesic, cytotoxics Moduretic and diazepam, than in the remaining cases (Table I). No drugs were associated with a lower metabolic ratio. In the 65 extensive metaboliser cases not taking any of the above four drugs, mean metabolic ratio was statistically significantly lower than in the 95 extensive metaboliser controls (none of whom were taking any of the four drugs) (Table I).

This study confirms that fewer lung cancer cases than controls were poor metabolisers of debrisoquine (1.9% *vs* 8.7%). The results of Ayesh *et al.* (1984) were very similar; four of 245 (1.6%) cases and 21 of 234 (9.0%) controls were poor metabolisers ($P<0.01$). Poor metabolisers thus have one-fifth the risk of smoking-related lung cancer of extensive metabolisers. Among extensive metabolisers one might expect more lung cancer cases than controls to be homozygous, and in keeping with this metabolic ratio was lower on average (i.e. more debrisoquine was hydroxylated) in lung cancer cases (excluding those taking certain drugs) than in controls.

No apparent sources of bias can explain the association of extensive debrisoquine hydroxylation with lung cancer. Those drugs known to affect metabolic ratio increase it, the bias thus operating against the result of fewer poor metabolisers among lung cancer cases. A biological effect of the cancer is a possible explanation, but in animal studies mono-oxygenase activities appear depressed by the presence of cancer (Rosso *et al.*, 1971), such a bias again operating against the result. Steiner *et al.* (1985) found that smoking and various other environmental factors such as alcohol, body weight and exercise that might differ between cases and controls did not demonstrably influence metabolic ratio, apart from a modest association with coffee intake. Moreover, as discussed by Nebert (1981), the metabolic effects of environmental factors are generally not powerful enough to mimic genetic effects of the magnitude observed here (extensive metabolisers hydroxylating about 50 times as much debrisoquine as poor metabolisers on average). We conclude that the association observed in two studies, in the absence of any apparent source of bias, constitutes evidence for genetic predisposition to smoking-related lung cancer. The association may be interpreted directly as a shared enzymatic pathway with an unspecified carcinogen, or indirectly as linkage disequilibrium, the gene locus regulating debrisoquine hydroxylation being closely associated on the same chromosome with a locus that independently affects lung cancer risk.

Further progress in elucidating metabolic predisposition to cancer is likely to be dominated by recombinant DNA technology, which is free from the bias associated with metabolic studies. The gene responsible for extensive debrisoquine hydroxylation is a structural gene, its complementary DNA has been cloned and sequenced, and three different mutant genes have been identified that give rise to incorrectly spliced messenger RNAs unable to yield an immunodetectable protein in the liver (Gonzalez *et al.*, 1988). Unfortunately these three mutant genes can account for only about half of all poor metabolisers, so that molecular genetics cannot at present replace the pharmacological measure of debrisoquine metaboliser status.

We thank the clinicians of the Brompton Hospital, London, for permission to study their patients. The debrisoquine assays were performed by Dr Timothy P. Sloan, who was sadly lost in an accident in 1983.

### References

AYESH, R., IDLE, J.R., RITCHIE, J.C., CROTHERS, M.J. & HETZEL, M.R. (1984). Metabolic oxidation phenotypes as markers for susceptibility to lung cancer. *Nature*, **312**, 169.

EICHELBAUM, M. (1984). Polymorphic drug oxidation in humans. *Fedn Proc.*, **43**, 2298.

EVANS, D.A.P., MAHGOUB, A., SLOAN, T.P., IDLE, J.R. & SMITH, R.L. (1980). A family and population study of the genetic polymorphism of debrisoquine oxidation in a white British population. *J. Med. Genet.*, **17**, 102.

GONZALEZ, F.J., SKODA, R.C., KIMURA, S. and 6 others (1988). Characterisation of the common genetic defect in humans deficient in debrisoquine metabolism. *Nature*, **331**, 442.

IDLE, J.R., MAHGOUB, A., ANGELO, M.M., DRING, L.G., LANCASTER, R. & SMITH, R.L. (1979). The metabolism of [$^{14}$C]-debrisoquine in man. *Br. J. Clin. Pharmacol.*, **7**, 257.

MILLER, J.A. & MILLER, E.C. (1983). The metabolic activation and nucleic acid adducts of naturally occurring carcinogens: recent results with ethyl carbamate and the spice flavours safrole and estragole. *Br. J. Cancer*, **48**, 115.

NEBERT, D.W. (1981). Possible clinical importance of genetic differences in drug metabolism. *Br. Med. J.*, **283**, 537.

ROOTS, I., DRAKOULIS, N., PLOCH, M. and 6 others (1988). Debrisoquine hydroxylation phenotype, acetylation phenotype, and ABO blood groups as genetic host factors of lung cancer risk. *Klin. Wochenschr.*, **66**, suppl. XI, 87.

ROSSO, R., DONELLI, M.G., FRANCHI, G. & GARATTINI, S. (1971). Impairment of drug metabolism in tumour-bearing animals. *Eur. J. Cancer*, **7**, 565.

SLOAN, T.P., MAHGOUB, A., LANCASTER, R., IDLE, J.R. & SMITH, R.L. (1978). Polymorphism of carbon oxidation of drugs and clinical implications. *Br. Med. J.*, **ii**, 655.

STEINER, E., ISELIUS, L., ALVAN, G., LINDSTEN, J. & SJOQVIST, F. (1985). A family study of genetic and environmental factors determining polymorphic hydroxylation of debrisoquine. *Clin. Pharmacol. Ther.*, **38**, 394.